# Comparison of Algorithms for Kinship Inference Using the Verogen ForenSeq^®^ Kintelligence Kit

**DOI:** 10.3390/genes17030357

**Published:** 2026-03-23

**Authors:** Ciara Di Scala, Kelly Grisedale, Jodie Ward, Dennis McNevin

**Affiliations:** 1 Centre for Forensic Science, School of Mathematical and Physical Sciences, Faculty of Science, University of Technology, Sydney, NSW 2007, Australia; ciara.discala@student.uts.edu.au (C.D.S.);; 2 Forensic Science South Australia, Adelaide, SA 5000, Australia; 3 School of Biological Sciences, College of Sciences, Adelaide University, Adelaide, SA 5000, Australia; 4 Forensic Human Identification Global Services Pty Ltd., Sydney, NSW 2228, Australia

**Keywords:** kinship analysis, forensic investigative genetic genealogy, Kintelligence, single nucleotide polymorphism

## Abstract

Background/Objectives: Forensic kinship analysis is a rapidly developing practice that uses genetic data to identify unknown persons of interest through their genetic relatives. It can be used to generate new leads in forensic investigations, especially those involving long-term missing persons and unidentified human remains. More recently, the advent of SNP profiling panels designed specifically for forensic use has led to the exploration of kinship analysis using medium-density SNP data. This study aimed to evaluate the extent to which genetic relationships could be inferred using such data, and to assess the performance of different kinship inference methods. Methods: Kinship analysis was performed with both real and simulated profiles using the panel of SNPs contained within the Verogen ForenSeq^®^ Kintelligence Kit, with a wide range of relationship types and seven types of kinship inference methods. Results: It was determined that kinship inferences were possible out to the fourth degree of kinship, and all inference methods analysed were equally effective when tested using simulated data. However, some variation between methods was observed when they were analysed using real sample data, suggesting that further study is needed using a larger sample size. Conclusions: The results of this study demonstrate that medium-density SNP data is sufficient for extended kinship inference out to the fourth degree, and that several kinship inference methods are suitable for use with the Verogen ForenSeq^®^ Kintelligence Kit. These findings will support its application in forensic investigations involving the inference of distant genetic relationships.

## 1. Introduction

Kinship analysis involves identifying the genetic relatives of an unknown person of interest through the analysis of genetic markers such as short tandem repeats (STRs) and single-nucleotide polymorphisms (SNPs) [1,2]. This approach can provide critical investigative leads, particularly in cases involving unidentified human remains and long-term missing persons [3,4,5,6]. It is also an essential aspect of forensic investigative genetic genealogy (FIGG), which is a rapidly expanding field involving the reconstruction of family pedigrees using both genealogical resources and kinship analysis. Enhancements in DNA sequencing technologies, such as the development of massively parallel sequencing (MPS), also known as next-generation sequencing (NGS), in combination with targeted library enrichment, have facilitated the use of large SNP multiplexes for kinship inference. The analysis of DNA profiles consisting of hundreds of thousands of SNPs (high-density SNP genotypes) from whole-genome sequencing (WGS) or microarrays makes it possible to detect genetic relationships as distant as third cousins, or the seventh degree of kinship [7].

The use of high-density panels for forensic kinship analysis can be prohibitive, however, due to the higher DNA input requirements (especially for microarrays) and the costly equipment needed to perform sequencing [8,9]. High-density panels also often include SNPs that can be used to infer medical information, and gleaning such information, even indirectly, could breach the privacy of an individual and their genetic relatives [10,11]. Medium-density panels such as the Verogen ForenSeq^®^ Kintelligence Kit (QIAGEN, Hilden, Germany) [12] and FORensic Capture Enrichment (FORCE) [13] panel sequence far fewer SNPs (approx. 10,000 and 5000 SNPs, respectively) than high-density genotyping methods (typically more than 500,000 SNPs for microarrays and many human enrichment panels), and as such require less input DNA than some sequencing techniques. They are also more accessible for forensic use since sequencing can be performed on platforms that many forensic laboratories already use.

Despite this reduction in SNP density, these kits are still capable of a high discriminating power when used to identify familial relationships up to second cousins [14,15,16,17,18,19,20]. They are designed for sequencing degraded samples typically recovered in forensic contexts and exclude medically informative SNPs that can be present in high-density SNP panels, thereby enhancing their suitability for forensic applications and minimising potential privacy risks.

Currently, kinship analysis algorithms are often accessed online, meaning investigators must upload SNP profiles onto potentially vulnerable servers [21,22,23]. There has been a recent notable data leak and privacy breach on one of these platforms [24]. It has been suggested that forensic agencies should develop their own algorithms for kinship analysis, which could be used in-house without the need for uploads to public databases so that privacy can be maintained, and the risk of data breaches can be minimised [25]. While previous studies have explored using the Verogen ForenSeq^®^ Kintelligence Kit (referred to as the Kintelligence Kit henceforth) for kinship analysis [14,15,16,17,18,19,20], little research has been done to design such a program for this SNP panel.

In this study, we aimed to evaluate a wide range of kinship analysis methods for their use as an in-house algorithm. Genetic relationships ranging from the 1st to the 9th degree of kinship and unrelated individuals were used to assess seven kinship inference methods, which were applied using two different kinship algorithms. The methods used included a novel method for kinship analysis, as well as principal component analysis (PCA) and principal coordinate analysis (PCoA), which, to our knowledge, have never been assessed as kinship inference methods for medium-density data. We used profiles simulated using the panel of SNPs contained in the Kintelligence Kit (Table 1), as well as real Kintelligence Kit profiles, to assess the suitability of various kinship inference tools for use in forensic investigations.

## 2. Materials and Methods

### 2.1. Coding Environment and Reference Data

All statistical analysis was performed using the R coding environment through RStudio version 4.3.0 [26]. SNP sequencing data from the 1000 Genomes Project Phase 1 [27] was acquired using the SPSmart portal v5.1.2, an online tool for retrieving data from various SNP genotype databases [28]. The SPSmart ‘Search by SNPs’ function was used to search for the 10,230 SNPs in the Kintelligence Kit, which yielded 10,030 SNPs. Only autosomal SNPs were used from the Kintelligence Kit, as data was not available for the X and Y chromosomes. SNP genotypes were downloaded from SPSmart for the Northern Europeans from Utah (CEU) and Finnish in Finland (FIN) populations to give a total of 180 profiles.

### 2.2. Profile Simulation for Algorithm Testing

The 180 profiles obtained from the SPSmart portal were combined into a variant call format (VCF) file. This file acted as the input for profile simulation using Ped-sim v1.4.1 [29]. Eighteen relationship types (not including unrelated) were simulated using Ped-sim for algorithm testing. For each relationship type, 100 pairs were simulated using the default simulation parameters of 10^−3^ per SNP for missingness and genotype error, and 0 for pseudo-haploid and opposite homozygous error. Simulations were performed using the sex-specific genetic map described by Bhérer et al. [30], as well as the crossover interference model [31,32]. Unrelated pairs were created by randomly pairing profiles from the SPSmart data, which only consisted of unrelated individuals.

Of the 100 pairs for each relationship, 50 pairs were used as reference samples for calculating algorithm metrics, including mean total segment lengths, mean gamma distributions and conditional probabilities, for each relationship type. The remaining 50 pairs were used for kinship inference testing of each algorithm. In addition to the simulated pairs, 45 pairs of profiles from a family of volunteers (referred to as the family data henceforth) were also used for algorithm testing. Ethics approval for the use of the family data for this research was granted under ETH24-10015 by the University of Technology Sydney (UTS) Human Research Ethics Committee. Further details on the family data can be found in Figure S1 and Table S1.

An additional 900 simulated pairs (50 per relationship type) were generated using only CEU individuals for PCA and PCoA analysis, with 50 unrelated pairs created by pairing random profiles from the CEU SPSmart data.

### 2.3. Kinship Algorithms

Kinship analysis methods can be grouped into two main categories: analysis of inherited long haplotypes (referred to as identical-by-descent (IBD) methods) or analysis of concordant genotypes or alleles (referred to as identical-by-state (IBS) methods) [33]. IBD methods rely on the detection of IBD segments, which are sections of DNA that are identical between two individuals due to inheritance from a recent common ancestor. These can be detected by finding regions of adjacent concordant SNP alleles within compared DNA profiles. IBS methods instead use allele states to calculate metrics such as the likelihood of the profile data given alternative hypotheses (likelihood ratio calculations), or the probability that two individuals share a randomly chosen allele by descent (kinship coefficient).

Four IBD-based and three IBS-based kinship inference methods were assessed. Each IBD method was analysed in combination with two IBD-segment detection algorithms, producing eight algorithm–inference method combinations. Each method was assessed for its suitability for kinship inference by plotting the distributions of the associated algorithm metric for each relationship type, or in the case of the gamma distribution method, for a limited subset of the relationships. An algorithm was considered suitable for kinship inference if there was differentiation of at least the 1st–3rd degrees of kinship when plotting the algorithm metric, as this was consistent with the minimum level of distinction previously shown to be achievable with the Kintelligence Kit [14]. Algorithms that did not demonstrate sufficient differentiation between degrees of kinship were deemed unsuitable and excluded from further analysis.

#### 2.3.1. IBD Segment Detection Algorithms

***Algorithm S1***

The first algorithm for IBD segment detection (referred to as Algorithm S1 henceforth) describes a workflow for identifying IBD segments, which first involves applying a ‘base’ algorithm that identifies stretches of concordant SNP genotypes shared by a pair of individuals Algorithm S1), referred to as IBS regions. The foundation of the base algorithm is a loop that iterates through each row of a pair of SNP profiles to identify whether the current pair of genotypes is the start or end of a run of concordant SNPs. It does so by identifying the first SNP with concordant genotypes after a non-concordant SNP and storing that position as the start of a new IBS region. It checks whether each following SNP is still concordant until non-concordant genotypes are encountered, which is then stored as the region end position. Once the entire genome is scanned, the function returns the starting and ending positions of all concordant regions, as well as their lengths in both million base pairs (Mbp) and number of SNPs.

Following this, filtering steps are applied to remove regions that are likely to be IBS but not IBD. The filtration method used was based on the protocol described by Schütz et al. [34] in their supplementary data. They describe a novel method for IBD-segment filtration that uses a ‘SNP informativity’ metric, $M_{I}(i)$, which is calculated using a count, $I_{c}$, of the number of times each SNP is included in an IBS region (Equation (1)):

(1) $$M_{I}\left(i\right)=\frac{med\left(I_{c}\right)}{I_{c}\left(i\right)},$$

where $I_{c}\;$ is the IBS count of SNP i, $med\left(I_{c}\right)$ is the median of all IBS counts and $M_{I}(i)$ is the informativity of SNP i. This metric is summed over the SNPs within an IBD segment to give the IBD informativity, $I_{s}(j)$ (Equation (2)), which is a measure of the likelihood of an IBS region being IBD:

(2) $$I_{s}\left(j\right)=\sum_{i\in M\left(j\right)}M_{I}\left(i\right),$$

where M(j) is all markers within segment j. We used an altered version of this method where, in the first step, IBS regions < 10 Mbp in size were filtered from the data. An IBD informativity threshold of 50 was then used to filter any IBS regions from the remaining segments. In addition, we adapted the secondary step of the method employed by Schütz et al. [34], which trims filtered segments to remove sections that overlap with low-confidence genomic regions that were shown to give rise to erroneous IBD (referred to as masked regions). We performed the same function on our data, but using regions of the Kintelligence Kit where there were gaps in SNP coverage (these masked regions can be found in Table S2 and Figure S2). In this final step, sections of IBD segments that overlap with low-coverage regions were trimmed, and the remaining segment(s) were kept if they remained greater than 10 Mbp in length and still had an IBD informativity greater than 50.

***Algorithm S2***

The second algorithm for IBD segment detection (referred to as Algorithm S2 henceforth) describes an alternative workflow that uses a ‘windowed’ method to identify IBD segments, based on the protocol described in Snedecor et al. [14]. In this method, each chromosome in a pair of profiles is divided into overlapping sections (i.e., windows), where each window starts at an SNP S in the chromosome and extends to a length of L SNPs. Each subsequent window begins at the SNP after the starting SNP in the previous window and spans the same length L. This results in a set of windows W for each chromosome as follows:

$$W=\left\{S_{1},\ldots ,\;S_{1\;+\;L}\right\},\;\left\{S_{2},\;\ldots ,\;S_{2\;+\;L}\right\},\;\ldots ,\;\{S_{n},\;\ldots ,\;S_{n\;+\;L}\}$$

where $S_{1}$ is the first SNP in a chromosome and $S_{n\;+\;L}$ is the last. We used an SNP length (L) of 80 as the window size, which gave the most accurate kinship inference results. For each window, the kinship coefficient was calculated using Equation (7) (see Section 2.3.3 for Kinship Coefficient), and all windows with a kinship coefficient greater than 0.23 were stored. If these windows overlapped, they were merged into segments. Finally, the proportion (p) of each segment where at least one allele was shared per genotype was calculated, and segments with p < 0.95 were removed from consideration (as recommended by Snedecor et al. [14]). See Supplementary Materials for further details on the procedure used in Algorithm S2.

#### 2.3.2. IBD-Based Kinship Inference Methods

***Mean total segment lengths***

For each IBD-segment detection algorithm, the total shared segment length of each sample pairing was calculated by summing the length of all its detected IBD segments. The mean total segment length, $M_{S}$, for each relationship type was then found by averaging the total shared-segment lengths of all sample pairs that shared that relationship. Kinship inferences for each sample pairing were performed by subtracting their total shared-segment length individually from each $M_{S}$ to get the absolute difference between them. The relationship type with the smallest difference was the inferred relationship of the sample pairing.

***Gamma distributions***

The gamma distribution method is a novel technique based on the concept that IBD-segment length distributions can be approximated by gamma distributions because recombination, the underlying process responsible for forming IBD segments, can be modelled as a Poisson process. Poisson processes naturally give rise to gamma-distributed data, with the probability of an event occurring decreasing as an independent variable increases. In the context of IBD segments, this means that the more distant the relationship, the less likely it is for a pair of individuals to share longer segments and the more rapid the decay of the gamma distribution [35]. This property of IBD segments provides a novel avenue for kinship inference, as the gamma distributions that fit the IBD-segment lengths of different genetic relationships vary depending on how distant the relationship is.

Kinship inferences were performed by comparing gamma distributions of IBD-segments shared by a sample pairing to average distributions for each relationship type. For each IBD-segment detection algorithm, average gamma distributions were calculated using the IBD segments detected for each set of 50 pairings in the reference dataset. For each relationship type, all IBD segments detected were compiled into separate dataframes. Gamma distributions were fitted to each of these compiled dataframes using the R package fitdistplus (version 1.2.1) [36]. The function fitdist() was applied to the data using maximum likelihood estimation. The scale parameter, θ, of the gamma model was determined by dividing the mean length of IBD segments for each relationship type by the shape parameter, k, describing the distribution.

To obtain a set of values that could be used to compare gamma distributions, Equation (3) was used to calculate the probability of an IBD segment of length x, where x was every integer in the range 1–150 Mbp (the maximum length of segments observed in the Algorithm S1 data). When this equation is used to find the probability of a range of IBD-segment lengths, it produces a frequency distribution of expected IBD-segment lengths shared by two individuals who are genetically related, where Γ is the gamma function:

(3) $$P\left(x\right)=\;\frac{1}{\Gamma (k)\theta^{k}}x^{k-1}e^{\frac{-x}{\theta }}$$

This was done for both the average distributions and the sample pairing distributions. Equation (4) was used to compare the gamma distribution of a sample pairing against the average gamma distributions for each relationship type by summing the absolute difference between each P(x) and the corresponding average P(x), and dividing by 150 (the number of IBD-segment lengths, x, in the range 1–150 Mbp). For each relationship type, the average absolute difference was found, and the relationship with the lowest average was considered the inferred relationship. Note that gamma distributions could only be modelled for pairings that shared at least two segments.

(4) $$M_{p,r}=\frac{1}{150}\sum_{x\;\in \;R}abs({P\left(x\right)}_{p}-{P\left(x\right)}_{r}),$$

where $M_{p,r}$ is the mean difference between the gamma distributions of pairing p and relationship r, and R is every number in the range 1–150 Mbp.

***Conditional probability***

The conditional probability method is a simplified version of the kinship inference protocol described by Ancestry.com in their white paper [37]. It involves determining the probability of a relationship (R) given the total length of segments shared IBDaccording to Equation (5):

(5) $$P\left(R|S\right)=P\left(R⋂S\right)/P(S)$$

To determine $P\left(R|S\right)$, the range of possible total shared segment lengths (0–3000 Mbp) was divided into intervals of 50 Mbp. These intervals acted as a discrete measure of the total amount ofDNA shared IBD by each sample pairing, and represent S in Equation (5). For each interval S and each relationship type R, $P\left(R⋂S\right)$ was found by counting the number of pairings that had a total shared-segment length within the interval. This number was then divided by the total number of pairings of any relationship type within each interval, i.e., P(S). For each sample pairing, the relationship with the greatest probability within their interval was the relationship they were inferred to share.

***IBD0 proportion***

The underlying assumption of the IBD0 proportion method of kinship inference is that the proportion of the genome in which two individuals do not have detectable IBD sharing aligns with the probability of zero IBD sharing ($\pi_{0}$) given their genetic relationship. Given this assumption, finding the IBD0 proportion of a sample pairing is a matter of simply subtracting the total length of the shared segments from the length of the genome. Following this, kinship inferences were found using the inference ranges provided by Manichaikul et al. [38] (Table 2), expanded to include the 4th–9th degrees of kinship.

#### 2.3.3. IBS-Based Kinship Inference Methods

***Kinship Coefficient***

The kinship coefficient is defined as the probability that a pair of individuals will share a randomly chosen allele by descent, and is generally given by Equation (6):

(6) $$\phi =\;\frac{k_{1}}{4}+\frac{k_{2}}{2}$$

where $k_{1}$ and $k_{2}$ are the fractions of the genome that two individuals share that are IBD1 and IBD2, respectively [39]. Since the IBD-segment detection algorithms used in this study do not separate IBD1 and IBD2 regions, we used the robust kinship estimator given by Manichaikul et al. [38], which uses the IBS states of individuals i and j to estimate their kinship coefficient according to Equation (7):

(7) $$\phi =\;\frac{N_{Aa,Aa}-2N_{AA,aa}}{2N_{Aa}^{\left(i\right)}}+\frac{1}{2}-\frac{1}{4}\frac{N_{Aa}^{\left(i\right)}+N_{Aa}^{\left(j\right)}}{N_{Aa}^{\left(i\right)}}$$

where $N_{Aa,Aa}$ is the number of SNPs in which i and j are both heterozygous, $N_{AA,aa}$ is the number of SNPs where i and j are opposite homozygotes, $N_{Aa}^{\left(i\right)}$ is the number of SNPs where i is heterozygous, and $N_{Aa}^{\left(j\right)}$ is the number of SNPs where j is heterozygous. Note that i should be whichever individual has greater heterozygosity.

Equation (7) was used to calculate the kinship coefficient for each of the sample pairings, and their inferred relationship was determined using the inference ranges from Manichaikul et al. [38] (expanded to include 4th–9th degree relatives) in Table 3.

***PCA and PCoA***

While PCA-based kinship inference methods have been published previously, such as PC-Relate [40], using PCA/PCoA to directly infer kinship for medium-density SNP data has, to our knowledge, not yet been explored. To perform PCA, the 950 CEU simulated pairs were reformatted into a genotype matrix, which contained one row per pairing and one column per SNP. In each cell, the number of alleles that a particular pairing shared that were IBS for a particular locus was stored as a value of 0, 1 or 2, with missing genotypes encoded using −1. The base R function prcomp() was applied to the matrix file, which gave a table of principal component values.

A similar process was applied for the PCoA analysis, in which the same genotype matrix was used but with missing genotypes encoded as ‘NA’ values. The dist.gene() and pcoa() function from the ape package (v5.8.1.) [41] were applied to the genotype matrix using the pairwise method to find the principal coordinates.

### 2.4. Algorithm Comparison

Algorithms deemed suitable for further testing were first compared by their accuracy rates for kinship inference when tested using both simulated and real data. When calculating accuracy rates, kinship inferences were considered correct if the inferred relationship was within the same degree as the known relationship, as distinguishing relationships within the same degree is often not possible for most algorithms and degrees of kinship without considering non-autosomal data [42].

To compare the efficacy of each algorithm, receiver operator characteristic (ROC) curves were also generated using the pROC package (v1.19.0.1.) [43] in R for the simulated data only. For each inference method, we classified inferences as either correct or incorrect depending on whether the inference was consistent with the known relationship type of a pairing of individuals. The performance of each method was measured by setting thresholds ranging from 0 to 5 degrees, where each threshold value represented an acceptable margin of error for inferences. For instance, a threshold value of 2 meant that any inference that was within ±2 degrees of the known pairing relationship was considered correct. These classifications were used to calculate the true-positive (sensitivity) and false-positive (1 − specificity) rates, as well as the area under the ROC curve (AUROC), so that inference methods could be compared.

## 3. Results

### 3.1. Algorithm Suitability for Kinship Inference

#### 3.1.1. IBD Methods

**Figure 1:**
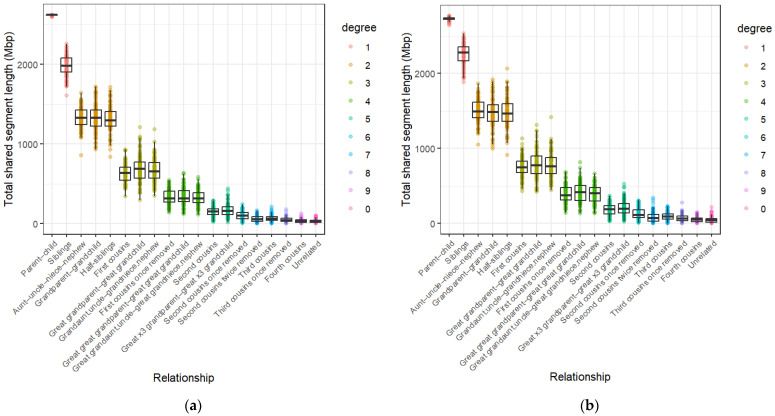
Total shared-segment length distributions for (**a**) Algorithm S1 and (**b**) Algorithm S2 across relationship types ranging from 1st to 9th degree of kinship, and unrelated individuals. Each point represents the total shared-segment length of a pairing of individuals.

**Figure 2:**
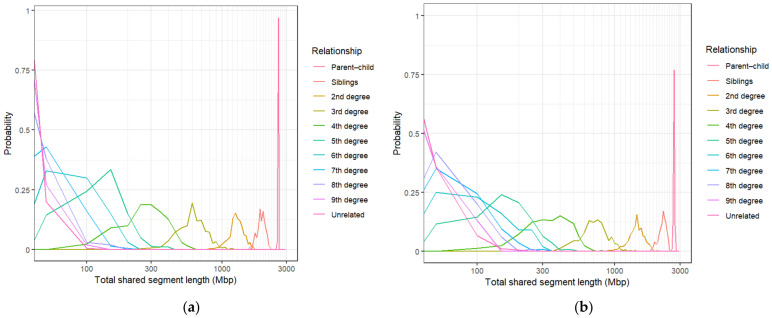
Conditional probability distributions of total shared-segment lengths given relationship type for (**a**) Algorithm S1 and (**b**) Algorithm S2. Note that the x-axes are shown in a logarithmic scale for better separation of distant (>4th degree) relationship distributions.

**Figure 3:**
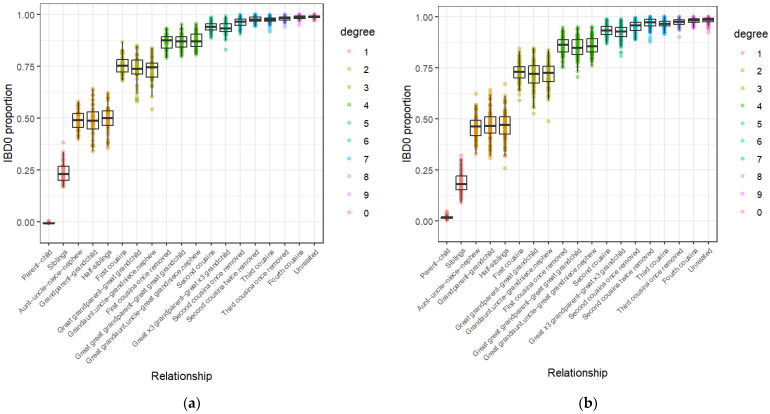
IBD0 proportion distributions for (**a**) Algorithm S1 and (**b**) Algorithm S2 across relationship types from the 1st to the 9th degree of kinship, and unrelated individuals (represented by degree = 0).

The distributions of the total shared-segment lengths for each relationship type for Algorithms S1 and S2 are given in Figure 1. The boxplots in each graph indicate that relationships within each degree of kinship have approximately the same median shared-segment length, although the Algorithm S2 distributions have slightly higher medians across all degrees of kinship compared to those from Algorithm S1. Despite these differences, significant differentiation amongst kinship classes seems to only be achievable up to the fourth degree of kinship for both algorithms. After this, distributions overlap by increasingly greater margins until they become indistinguishable from each other. The conditional probability (Figure 2) and IBD0 proportion distributions (Figure 3) show a similar pattern, with those of relationships beyond the fourth degree again overlapping considerably. Each of these methods seems to show sufficient differentiation of the 1st–4th degrees of kinship to be suitable for use as kinship inference methods.

The total shared-segment length results seem to largely align with expected values reported by the Shared cM Project [44] (which records self-reported total shared-segment lengths from various commercial DNA testing results) as well as simulated data from the literature [45]. The exception to this is the unrelated individuals, which seem to share an increased amount of IBD DNA according to the total shared-segment length and conditional probability data for both IBD algorithms (Figure 1 and Figure 2), but especially for Algorithm S2. Both methods show that while the majority of unrelated individuals share between 0 and 40 Mbp of their DNA IBD, some pairs share as much as 102 Mbp for Algorithm S1 and 214 Mbp for Algorithm S2. Since the data sourced from the 1000 Genomes Project consisted of only unrelated individuals, we can conclude that many of these segments are likely IBS, rather than IBD. This could indicate an underlying issue that may cause inaccuracies when distinguishing unrelated individuals from distant relatives.

To determine if this was an issue with the IBD-segment detection methods used, additional filtration steps were applied to Algorithms S1 and S2 segment data. We ran Algorithm S1 with additional masking for unrelated individuals in the testing dataset to remove segments that overlapped with the increased sharing regions identified (exact locations of updated masked regions can be found in Table S3). It was found that after applying this additional masking, 17.7% of unrelated individuals were still reported as sharing >50 Mbp (compared to 25.3% previously), with 77.7% sharing >0 Mbp. The total number of segments detected did decrease from 264 to 221, and the average segment size was reduced from 14.3 Mbp to 12.9 Mbp. Thus, it appears that additional masking did reduce some of the sharing reported for unrelated individuals but did not account for the majority of the increased sharing detected for Algorithm S1. We also attempted running Algorithm S2 with the filtering steps applied to Algorithm S1 (as detailed in Section 2.3.1, to determine if the original parameters used for Algorithm S2 were insufficient for filtering IBS regions. However, this also did little to reduce the IBD sharing for unrelated individuals, with the average segment size reducing from 48.9 to 36.1 Mbp.

**Figure 4:**
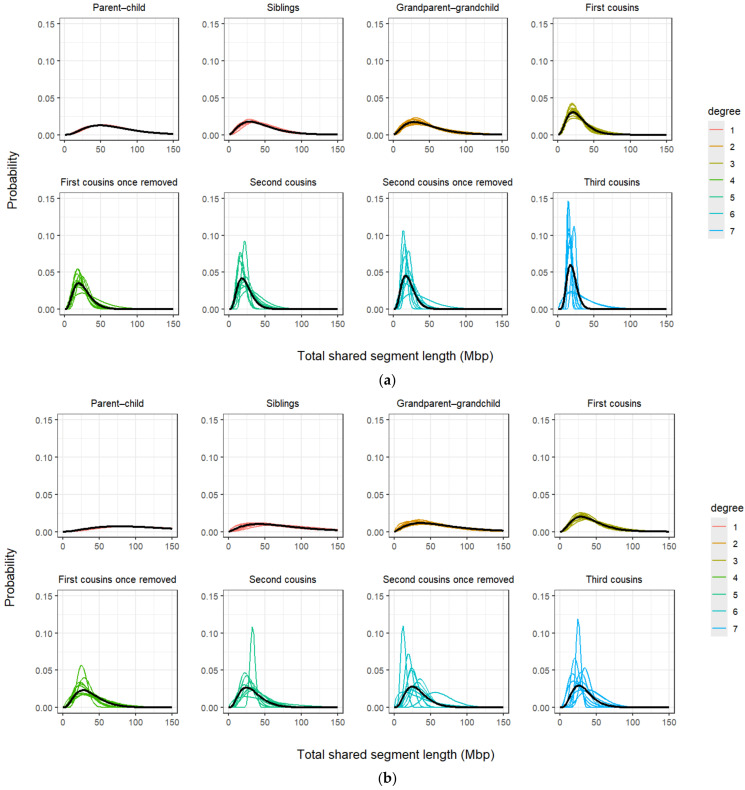
(**a**) Algorithm S1 and (**b**) Algorithm S2 gamma distributions for reference pairings from eight relationship types, with average distributions shown in black.

For the gamma distribution method to be suitable for kinship inference, there needs to be significant differences between the distributions of tested relationship types. Speed and Balding [35] postulate that the distributions of IBD-segment lengths decay more rapidly as relationships become more distant, which appears to be corroborated by simulation data analysed in this study. Figure 4a demonstrates that distantly related individuals (such as those in the sixth and seventh degrees of kinship) show IBD-segment distributions that often have a sharp peak at approximately 15 Mbp, which then rapidly drops off. In Figure 4b, these distributions have a much less distinct peak, suggesting that distantly related individuals have greater amounts of IBD sharing on average in the Algorithm S2 results. In comparison, those in the first and second degrees of kinship for both algorithms have fairly smooth distributions with gradual decay. The variation within each degree of kinship also appears to increase with genetic distance, so that the average IBD distributions (in black) resemble fewer of the sample distributions for more distant relationships. This implies that while many distantly related individuals may share similar amounts of their DNA IBD, the number and length of the segments shared vary markedly.

It also suggests that the gamma distribution method may not be able to accurately infer kinship, as many samples may have distributions that differ too greatly from the correct average distribution for this measure to act as an inference tool. Combined with the only minor differences between the average distributions of first–second and third–fourth degree relationships, respectively, the gamma distribution method may not be suitable for further analysis.

#### 3.1.2. IBS Methods

**Figure 5:**
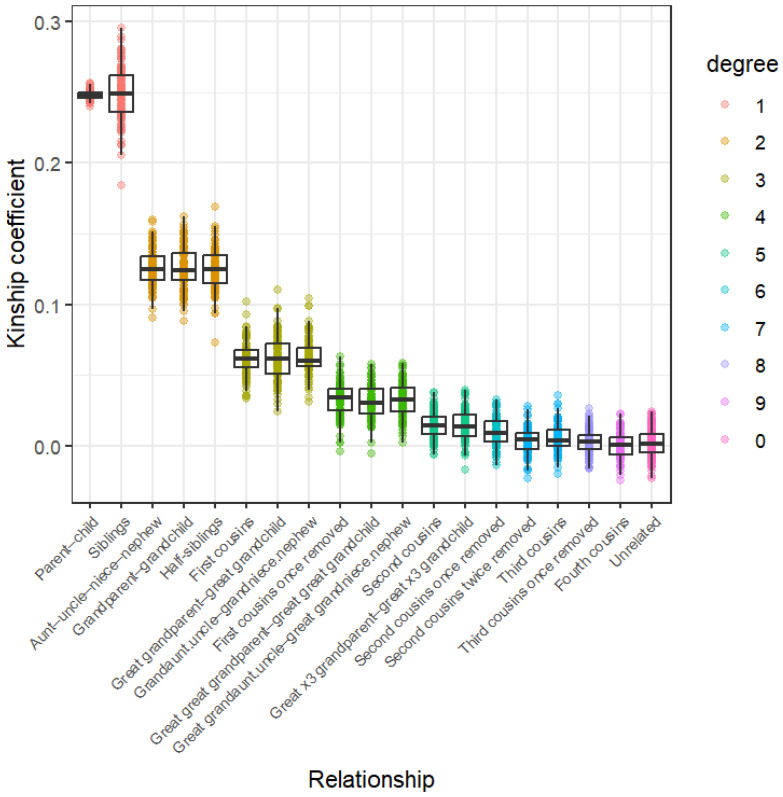
Kinship coefficient distributions across relationship types from the 1st to the 9th degree of kinship, and unrelated individuals.

The kinship coefficient method results appear very similar to those of the total shared-segment length method, in that relationship types within each degree have similar distributions, with those beyond the fourth degree of kinship showing increasingly large amounts of overlap (Figure 5). In this case, however, parent–child and sibling pairs cannot be easily distinguished, which is a feature of the kinship coefficient method. Both relationships within the first degree have almost identical median values, but siblings have a distribution with greater variance, while parent–child pairing coefficients only vary by approximately ±0.025. The differentiation between kinship coefficients for each degree of kinship seems adequate to allow for kinship inference analysis.

**Figure 6:**
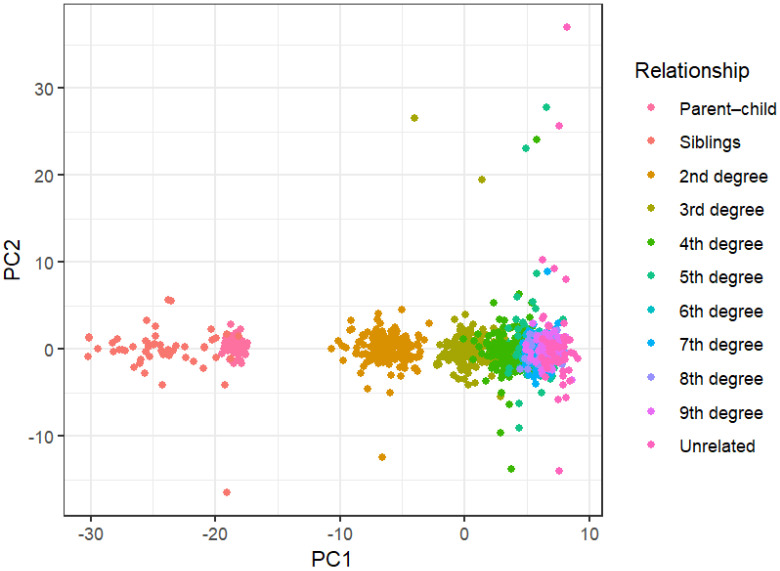
Principal component analysis (PCA) results for relationships from 1st to 9th degree, and unrelated individuals.

**Figure 7:**
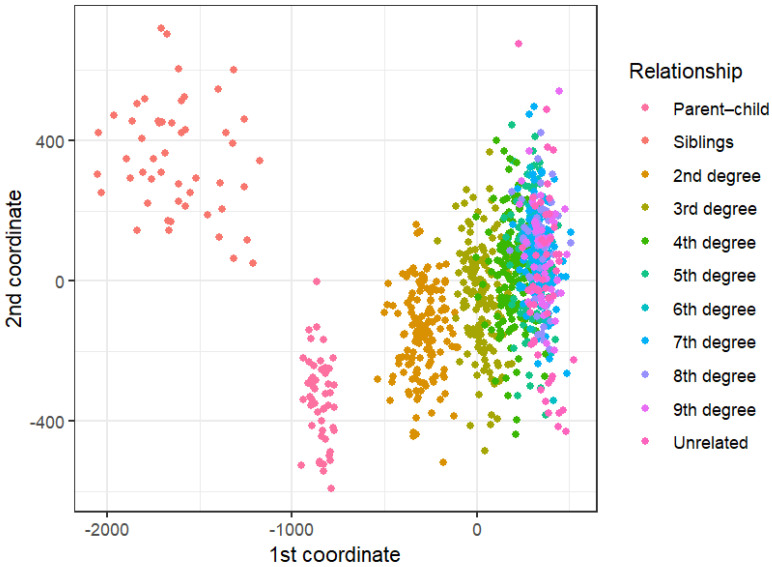
Principal coordinate analysis (PCoA) results for relationships from 1st to 9th degree, and unrelated individuals.

Figure 6 shows the PCA results for the 950 CEU samples. Moving from left to right across PC1, relationships starting from least (first degree) to most (unrelated) genetically distant appear to form a cline, with those in the first and second degree displaying the most differentiation. Unexpectedly, siblings appear to form the most distinct cluster on the far left, with parent–child pairs, which are usually the most easily distinguishable using other methods, forming a cluster closer to that of the second-degree relatives. Following this group, the third-degree pairs form their own cluster separate from the second degree, but which overlaps substantially with the fourth-degree pairs. All other relationship types are in a large grouping on the right that cannot be easily separated by degree of kinship. The PCoA plot (Figure 7) shows similar patterns to those of the PCA, in that the sibling pairs are the most distinguishable cluster on the upper-left-most side, followed by parent–child and second-degree pairs. The third- and fourth-degree pairs can again be seen forming overlapping groupings following this, with the remaining related individuals clustered tightly together on the right side.

The lack of differentiation between degrees of kinship beyond the second degree in the PCA and PCoA analysis signifies that these methods are not capable of sufficiently capturing pedigree-wide genetic similarity using medium-density SNP data. As such, these methods were abandoned for kinship inference testing.

### 3.2. Comparison of Kinship Inference Methods

#### 3.2.1. Simulated Data Inference Results

**Figure 8:**
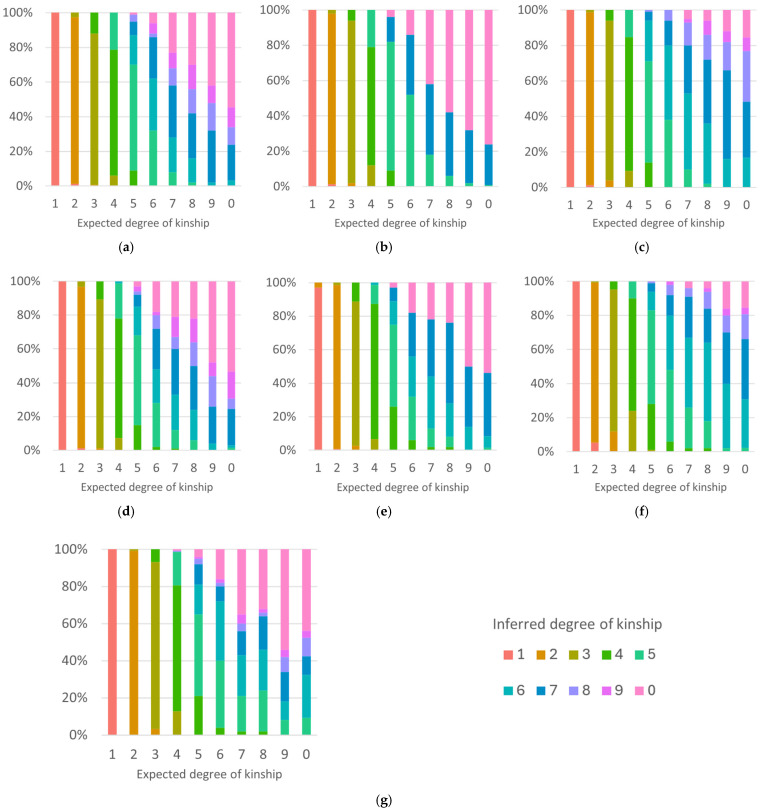
The kinship inference testing results for the (**a**) Algorithm S1 mean total segment length method; (**b**) Algorithm S1 conditional probability method; (**c**) Algorithm S1 IBD0 method; (**d**) Algorithm S2 mean total segment length method; (**e**) Algorithm S2 conditional probability method; (**f**) Algorithm S2 IBD0 method; and (**g**) kinship coefficient method for simulated data.

Figure 8 displays the results of kinship inference testing on the methods that were able to differentiate between at minimum the 1st–3rd degrees of kinship. All methods have close to 100% accuracy for the first and second degrees of kinship, with accuracy rates of 83–91% for the third degree of kinship, and 66–81% for the fourth degree. For the first four degrees of kinship, the majority of incorrect inferences are within one degree of the correct inference. Following this, the accuracy drops off to 44–73% at the fifth degree, and incorrect inferences are more often greater than one degree from the correct relationship, suggesting that inferences are less accurate and reliable beyond the fourth degree.

The ability to differentiate unrelated from related individuals is not ideal for most methods, with all except the Algorithm S1 conditional probability method having a maximum accuracy rate of 55%. This is likely a result of the number of unrelated individuals sharing >20 Mbp (62.3% and 76.9% of the Algorithms S1 and S2 test samples, respectively), making them harder to distinguish from distantly related pairs. This issue can be negated by redefining the classification parameters so that those classed as sixth degree or greater are considered unrelated, which increased the accuracy rate to a minimum of 91% for unrelated pairs.

While the gamma distribution method did not pass the assessment for its suitability for kinship inference, it was still tested as an inference method to determine the extent to which it could accurately infer kinship using both Algorithms S1 and S2. Its ability was extremely limited for both algorithms, with accuracy for the first degree being only 69% for Algorithm S1 and 80% for Algorithm S2. The results for this method can be found in Figure S3.

#### 3.2.2. ROC Curves

**Figure 9:**
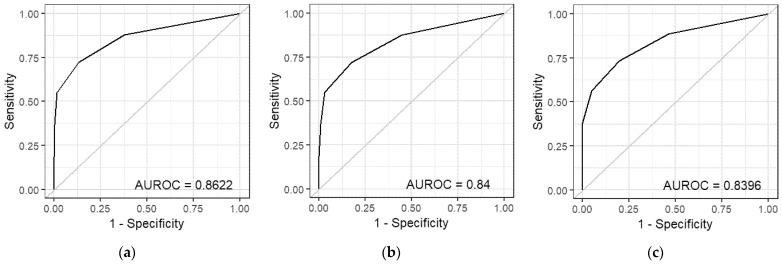

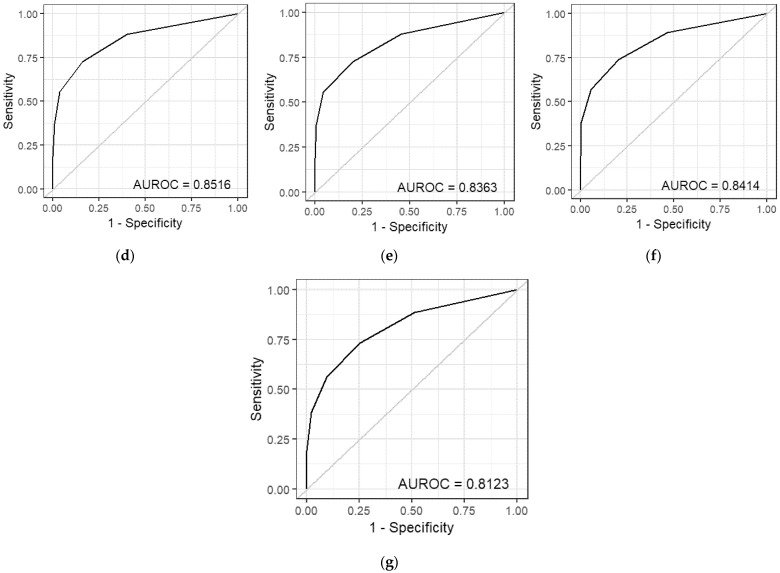
Receiver operator characteristic (ROC) curves for the (**a**) Algorithm S1 mean total segment length method; (**b**) Algorithm S1 conditional probability method; (**c**) Algorithm S1 IBD0 method; (**d**) Algorithm S2 mean total segment length method; (**e**) Algorithm S2 conditional probability method; (**f**) Algorithm S2 IBD0 method; and (**g**) kinship coefficient method.

ROC curves illustrate the compromise between increasing sensitivity and decreasing specificity as threshold values vary. In our analysis, we adjusted the acceptable margin of error for kinship inferences from 0 degrees to 5 degrees, which increased the number of pairs whose relationship was correctly inferred (true positives), while also decreasing the specificity with which relationships were classified. The ROC curves for each kinship inference method in Figure 9 are very similar, with AUROC values ranging from 0.8123 to 0.8622. As such, there appears to be no major differences between the classification accuracy of any of the inference methods when used for kinship inferences of simulated pairs of individuals.

#### 3.2.3. Family Data Inference Results

**Figure 10:**
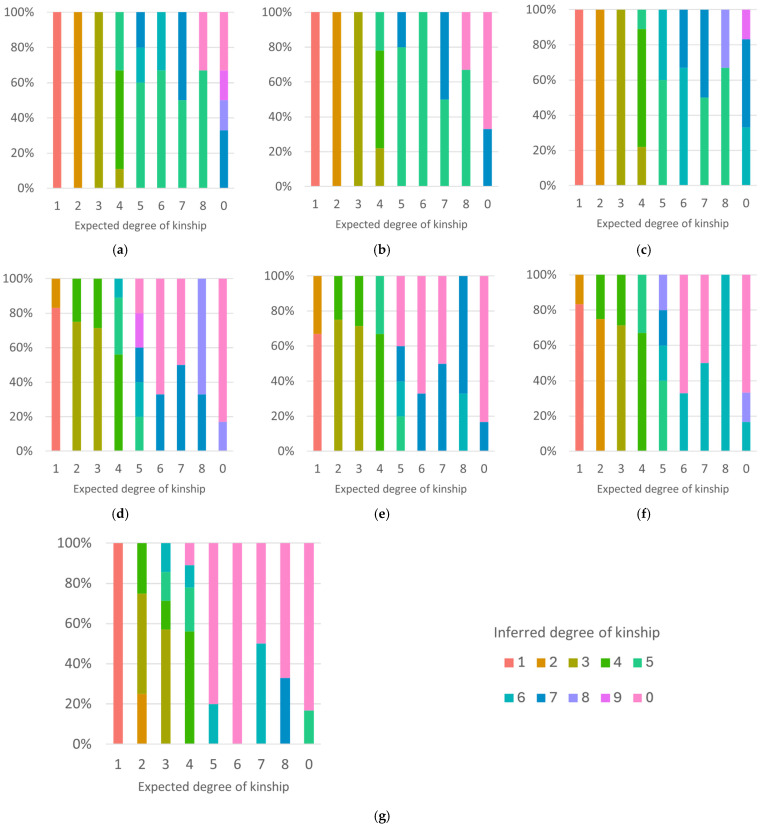
The kinship inference testing results for the (**a**) Algorithm S1 mean total segment length method; (**b**) Algorithm S1 conditional probability method; (**c**) Algorithm S1 IBD0 method; (**d**) Algorithm S2 mean total segment length method; (**e**) Algorithm S2 conditional probability method; (**f**) Algorithm S2 IBD0 method; and (**g**) kinship coefficient method for family data.

The kinship inference results using the family data showed greater differences in the performance of each kinship analysis method, especially when comparing the Algorithm S1 results with those of the Algorithm S2 and IBS methods (Figure 10). As was the case when using simulated data, none of the inference methods were able to reliably infer kinship beyond the fourth degree, with an average accuracy rate of 43% for the fifth degree. Accuracy rates for the 1st–4th degrees for the Algorithm S1 IBD inference methods were fairly similar to the simulated data, but with somewhat diminished inference accuracy rates for the fourth degree for the mean total segment length and conditional probability methods.

In comparison, the Algorithm S2 results show far less consistent inference capabilities across all inference methods, despite these methods having shown comparable accuracy rates to Algorithm S1 when tested using simulated data. This is especially apparent for the mean total segment length and conditional probability methods, and, in particular, for pairs in the second degree of kinship, which were misclassified at a much higher rate than expected. The kinship coefficient method also showed a notable decrease in accuracy, particularly for the second- and third-degree relationships. Small changes in accuracy rates, as seen for the Algorithm S1 IBD methods, can likely be attributed to the much smaller sample size of the family data, but larger changes may suggest further analysis is needed using a greater number of samples.

## 4. Discussion

Comparison of the kinship inference methods tested indicates that each of them can infer 1st–4th-degree relationships and perform with approximately the same accuracy according to the ROC curve analysis. When only the ROC curve and simulated data testing is considered, it appears that any of these methods would be sufficient for performing kinship inferences using data from the Kintelligence Kit. However, the kinship coefficient method, while having similar accuracy rates to the other methods, cannot be used to differentiate between parent–child and sibling relationships unless the individual κ coefficients are also calculated, and so may be less beneficial when that level of resolution is required. In comparison, the gamma distribution method had much lower accuracy rates for all degrees of kinship than any of the other methods (Figure S3). The gamma distributions are theoretically different for different kinship classes, but ultimately the amount of variation in gamma distributions within each degree of kinship was too great, and the amount of variation between degrees of kinship too little, for it to be used to infer relationships. In addition, the gamma inference method only works for pairs that share at least two segments, and as such it is ineffective for some distant relationships beyond the fourth degree of kinship.

While testing using simulated data showed that all methods were largely similar in their kinship inference accuracy, some differences did arise when testing was performed on the family data samples. In particular, the Algorithm S1 methods showed a decrease in accuracy for fourth-degree relationships, and the Algorithm S2 and IBS methods had diminished accuracy rates across the 1st–4th degrees. This suggests that the Algorithm S1 methods may perform better for real samples. However, given the small number of sample pairs available from the family data, future research with a greater number of real Kintelligence Kit samples may be needed to assess the accuracy of the methods tested in this study when used for real world applications.

Additional research may also be required to investigate the increased IBD sharing observed for unrelated individuals. In our analysis, we identified an increased amount of DNA shared IBD by unrelated individuals for both Algorithms S1 and S2, which made it more difficult to distinguish them from those in the 5th–9th degrees of kinship. Despite both algorithms applying filtration methods to remove segments likely to be IBS, many unrelated individuals were still reported as sharing a non-zero amount of IBD DNA, with some having as many as seven segments detected. It is possible that these false positives could be attributed to erroneous IBD regions, or IBD ‘hotspots’, which are often reported as regions with low recombination rates or low SNP density in proximity to centromeres and telomeres [34,46,47,48,49]. Mapping these segments did identify some regions of increased IBD sharing in our data; however, only some of these coincided with centromeres or regions previously identified in the literature (Figure S2).

Increased background relatedness is another potential cause of IBD DNA detected in unrelated individuals. It was reported by Frazer et al. [50] that, on average, unrelated individuals from the same population share 0.5% of their genome through recent IBD. Dimitromanolakis et al. [48] demonstrated that this percentage was even higher for populations such as that of Puerto Rico, where the average sharing amongst unrelated individuals was 28.5 centimorgans (cM). In comparison, the average sharing for the CEU and FIN populations is 2.37 cM and 16.1 cM, respectively. This may explain some of the excess DNA shared IBD detected in our data, particularly for those that only shared one or two short segments, but does not account for those with IBD sharing in excess of 50 Mbp.

This seems to suggest that either long stretches of genotype concordance are more likely to occur in the CEU and FIN populations than previously thought, or another phenomenon is the cause of the increased sharing amongst unrelated pairs. For instance, manual comparison of detected segments with those reported by Ped-sim for related individuals indicated that the IBD-segment identification algorithms used in this study tend to overestimate the size of shared segments. This overestimation can occur as SNP profile density lowers, due to the larger gaps between SNPs causing lower specificity in determining the start and end points of segments. It is likely this is contributing, at least somewhat, to the excessive IBD sharing reported for unrelated individuals. However, the extent to which this is the case cannot be verified, as the true locations of any IBD segments present in these unrelated pairings are unknown. It is also possible that the method of Schütz et al. [34] is less effective when used on medium-density SNP data, leading to more false positives and greater IBD sharing reported for unrelated individuals.

There is also evidence to suggest that the overestimation of IBD sharing for unrelated individuals is not an uncommon phenomenon when using the Kintelligence Kit for kinship inference. Watson et al. [17] found that unrelated individuals were often falsely identified as genetic relatives in GEDmatch™ database searches using Kintelligence Kit profiles, and Snedecor et al. [14] found an unrelated profile was reported as sharing 152 cM with their sample profile in a GEDmatch™ search. This may suggest that the Kintelligence Kit panel is susceptible to increased false positives when kinship inferences are performed using some algorithms due to increased IBD sharing estimates, which may explain why applying additional filtering steps did little to improve the increased sharing. As such, it may be necessary to treat some inferences with caution if their total shared-segment length is less than a certain threshold, as they may be unreliable.

From the kinship analysis results, we can conclude that kinship inferences are possible, at most, out to the fourth degree using medium-density SNP coverage. Beyond this, kinship inference accuracy rates for most methods assessed in this study decreased below 60%, and incorrect inferences were often greater than one degree of kinship from the correct relationship type. This conclusion supports the findings of many studies on kinship analysis using medium-density data [14,16,20,51], but does not align with others that report accurate (>80% correct) kinship inferences for fifth-degree relationships [15,52]. This could be a result of the greater number of samples used in these studies, compared to only 50 per relationship type used in this study. For future work, the number of sample pairings should be increased to determine if this has an impact on kinship inference accuracy. It may also be the result of these studies using a more limited range of 5–9 relationship types when testing inference models, compared to the 19 types we have used. While relationship types within the same degree generally share similar amounts of DNA IBD, some have greater amounts of variation that could make them more difficult to infer, leading to lower accuracy rates.

As more relationships are analysed, the amount of variation within each degree of kinship increases, making it more difficult to discern between kinship classes. Using a broader range of relationships is likely to produce a dataset that is more representative of the type of variation that may be observed in real-world scenarios, and therefore, the accuracy rates reported here are likely reflective of those that would be encountered during operational casework. Including an even greater range of relationship types, for instance, half-cousins and double-cousins, may make accurate kinship inferences even more challenging and could help identify relationship types that are difficult to infer using current methods. In addition, profiles generated from low-quality or -quantity DNA, such as those that may be encountered in forensic investigations, may result in a further reduction in inference accuracy depending on the sequencing technology and analytical approach used [9,14,53].

Kinship inferences are possible beyond the fourth degree using higher-density SNP profiles, but some studies show that there is a theoretical limit to kinship analysis that prevents any significant increase in accuracy with an increase in genetic data. This theoretical limit can be attributed to the decreasing amount of DNA shared IBD with increasing genetic distance between two individuals, such that relationships beyond the fifth degree have an increased probability of sharing no IBD DNA as they get more distant. Studies by Kruijver [45] and Donnelly [54] demonstrated this concept theoretically, and determined that by the ninth degree, there is a ~30% probability of no IBD sharing between related individuals. Since no kinship inference algorithm is completely accurate, and many are unable to detect very small segments that distantly related pairs may share, the number of distant relatives that cannot be detected is often even greater than the percentage that share none of their DNA by descent.

This theoretical limitation is often reflected in the results of other studies, which show that beyond the fifth degree, fewer relationships can be inferred regardless of SNP density. For instance, Li et al. [46] found that when WGS data was used to infer kinship, the relatedness estimation program ERSA had greater than 95% power to detect relationships up to the fifth degree, but only 50% power for detecting relationships up to the eighth degree. In comparison, Seidman et al. [49] and Ramstetter et al. [55] determined that data containing ~500,000 SNPs could achieve a kinship inference accuracy of over 90% for third-order relationships, approximately 80% for fourth-degree and at most 69% for fifth-degree relationships for various inference methods, which is not dissimilar to the accuracy rates reported in this study. Tillmar and Kling [15] showed that 95 K and 560 K SNP panels both had ~50% accuracy for inferring seventh-degree relatives (third cousins), while the Kintelligence Kit had ~25% for the same degree.

These studies clearly indicate that, even when using the highest possible SNP density, the ability for kinship inference algorithms to detect relationships beyond the fifth degree is often limited. Hence, increasing SNP density past a certain point may give only a marginal benefit, especially in the context of FIGG. An increasingly extensive amount of genealogical research would be required to identify an unknown person of interest by their genetic relationships beyond the fifth degree of kinship, and in some cases, it may not be possible to resolve a case based only on very distantly related individuals [21,56,57]. For instance, identifying an unknown DNA donor by their third cousin may require investigating a pool of over 150 individuals [33,58], which could easily require constructing a pedigree of many hundreds of people [59], and may take upwards of 100 h of investigative work [57]. As such, medium-density data with in-house kinship algorithms presents a middle-ground that allows for extended kinship analysis while minimising issues that can arise when using higher-density SNP data alongside online kinship tools and databases.

## 5. Conclusions

Using a variety of relationship types from the 1st to the 9th degree of kinship, it was found that all of the inference methods showed sufficient accuracy for kinship inferences out to the fourth degree using simulated data. This is corroborated by the findings of some previous studies; however, others have reported higher kinship inference accuracy out to the fifth degree. The increased inference accuracy reported in these studies may be a result of using a reduced number of relationship types when testing inference methods, which is less representative of the variation one may encounter when using the Kintelligence Kit in practical forensic contexts. However, our Algorithm S1 methods, i.e., those that identified stretches of concordant SNP genotypes shared by a pair of individuals (IBS regions), performed better on Kintelligence Kit profiles from real samples compared with our Algorithm S2 methods (which used a ‘windowed’ algorithm to detect IBD segments) and the kinship coefficient method. In addition, we highlight the limitations of kinship analysis and suggest that increasing SNP density to improve the accuracy of kinship inferences may not provide significant benefit. As such, the capabilities of medium-density data, as reported in this study, may well be adequate for forensic purposes, offering a balance between genome coverage sufficient for extended kinship analysis and genetic privacy. These findings support the use of the Kintelligence Kit in forensic investigations, especially those involving the inference of distant genetic relationships, and its application alongside in-house kinship analysis algorithms. Future research on the Kintelligence Kit using a greater number of real samples and an even larger range of relationship types would assist in further establishing its limitations and uses for forensic kinship analysis.

## Figures and Tables

**Table 1:** The numbers and types of single-nucleotide polymorphisms (SNPs) in the Kintelligence Kit.

SNP Category	Number of SNPs
Kinship (kiSNPs)	9867
Ancestry (aiSNPs)	56
Identity (iiSNPs)	94
Phenotype (piSNPs)	22 *
X-SNPs	106
Y-SNPs	85
Total	10,230

* Two piSNPs are also classified as aiSNPs.

**Table 2:** The expected proportion of the genome with zero IBD sharing (IBD0) and inference ranges for various relationship types/degrees of kinship.

Relationship/Degree of Kinship	Expected IBD0 Proportion	Inference Ranges
Parent–child	0	≤0.1
Siblings	0.25	>0.1, ≤0.365
2	0.5	$>0.365,\;\leq 1-\frac{1}{2^{3/2}}$
3	0.75	$>1-\frac{1}{2^{\frac{3}{2}}},\;\leq 1-\frac{1}{2^{5/2}}$
4	0.875	$>1-\frac{1}{2^{\frac{5}{2}}},\;\leq 1-\frac{1}{2^{7/2}}$
5	0.9375	$>1-\frac{1}{2^{\frac{7}{2}}},\;\leq 1-\frac{1}{2^{9/2}}$
6	0.9688	$>1-\frac{1}{2^{\frac{9}{2}}},\;\leq 1-\frac{1}{2^{11/2}}$
7	0.9844	$>1-\frac{1}{2^{\frac{11}{2}}},\;\leq 1-\frac{1}{2^{13/2}}$
8	0.9922	$>1-\frac{1}{2^{\frac{13}{2}}},\;\leq 1-\frac{1}{2^{15/2}}$
9	0.9961	$>1-\frac{1}{2^{\frac{15}{2}}},\;\leq 1-\frac{1}{2^{17/2}}$
Unrelated	1	$>1-\frac{1}{2^{\frac{17}{2}}}$

**Table 3:** The expected kinship coefficients and inference ranges for various degrees of kinship. Note that a degree of 0 represents unrelated individuals.

Degree of Kinship	Expected Kinship Coefficient	Inference Ranges
1	$\frac{1}{4}$	$>\frac{1}{2^{5/2}},\;\leq \frac{1}{2^{3/2}}$
2	$\frac{1}{8}$	$>\frac{1}{2^{7/2}},\;\leq \frac{1}{2^{5/2}}$
3	$\frac{1}{16}$	$>\frac{1}{2^{9/2}},\;\leq \frac{1}{2^{7/2}}$
4	$\frac{1}{32}$	$>\frac{1}{2^{11/2}},\;\leq \frac{1}{2^{9/2}}$
5	$\frac{1}{64}$	$>\frac{1}{2^{13/2}},\;\leq \frac{1}{2^{11/2}}$
6	$\frac{1}{128}$	$>\frac{1}{2^{15/2}},\;\leq \frac{1}{2^{13/2}}$
7	$\frac{1}{256}$	$>\frac{1}{2^{17/2}},\;\leq \frac{1}{2^{15/2}}$
8	$\frac{1}{512}$	$>\frac{1}{2^{19/2}},\;\leq \frac{1}{2^{17/2}}$
9	$\frac{1}{1024}$	$>\frac{1}{2^{21/2}},\;\leq \frac{1}{2^{19/2}}$
0	0	$\leq \frac{1}{2^{21/2}}$

## Data Availability

The raw data supporting the conclusions of this article may be made available by the authors to approved entities upon written request and subject to consent provisions.

## Supplementary Materials

The following supporting information can be downloaded at https://www.mdpi.com/article/10.3390/genes17030357/s1: Figure S1: A pedigree showing the genetic relationships of the ten family members that provided a reference sample for this study. Algoritm S1: Pseudocode describing the workflow of the algorithm for identifying stretches of concordant SNP genotypes shares by a pair of individuals. Algoritm S2: Pseudocode describing the workflow of the algorithm for window-based IBD segment detection. Figure S2: All segments shared by unrelated test individuals for Algorithm S1 (a) and Algorithm S2 (b), mapped alongside the centromeres, increased sharing regions identified in the literature, and masked regions used in the IBD-segment detection and filtering algorithm. Figure S3: Results for the gamma distribution method of kinship inference for Algorithm S1 (a) and Algorithm S2 (b). ‘NA’ results indicate pairs whose relationship could not be inferred due to sharing less than two segments. Table S1: The genetic relationships shared by the family sample individuals from Figure S1. Table S2: Autosomal masked regions used in the identical-by-descent (IBD) detection and filtering algorithm, which also represent gaps in single-nucleotide polymorphism (SNP) coverage in the Verogen^®^ ForenSeq Kintelligence Kit. Table S3: Additional masked regions applied to the unrelated test pairings, which were identified by mapping segments shared by these pairs.

## Abbreviations

The following abbreviations are used in this manuscript:

STR	Short tandem repeat
SNP	Single-nucleotide polymorphism
FIGG	Forensic investigative genetic genealogy
MPS	Massively parallel sequencing
NGS	Next-generation sequencing
WGS	Whole-genome sequencing
PCA	Principal component analysis
PCoA	Principal coordinate analysis
CEU	Northern Europeans from Utah
FIN	Finnish from Finland
IBD	Identical by descent
IBS	Identical by state
Mbp	Million base pairs
ROC	Receiver operator curve
AUROC	Area under receiver operator curve
cM	Centimorgans
