# Supplementary material for: Comparison of Algorithms for Kinship Inference Using the Verogen ForenSeq® Kintelligence Kit
Source: Genes (Basel). 2026 Mar 23;17(3):357. doi: 10.3390/genes17030357 (PMC13026609; doi:10.3390/genes17030357)
Supplement: Supplementary file 1 [file genes-17-00357-s001.zip › genes-4188605-supplementary.pdf]

Supplementary Materials

Figure S1. A pedigree showing the genetic relationships of the ten family members that provided a reference sample for this study [1].

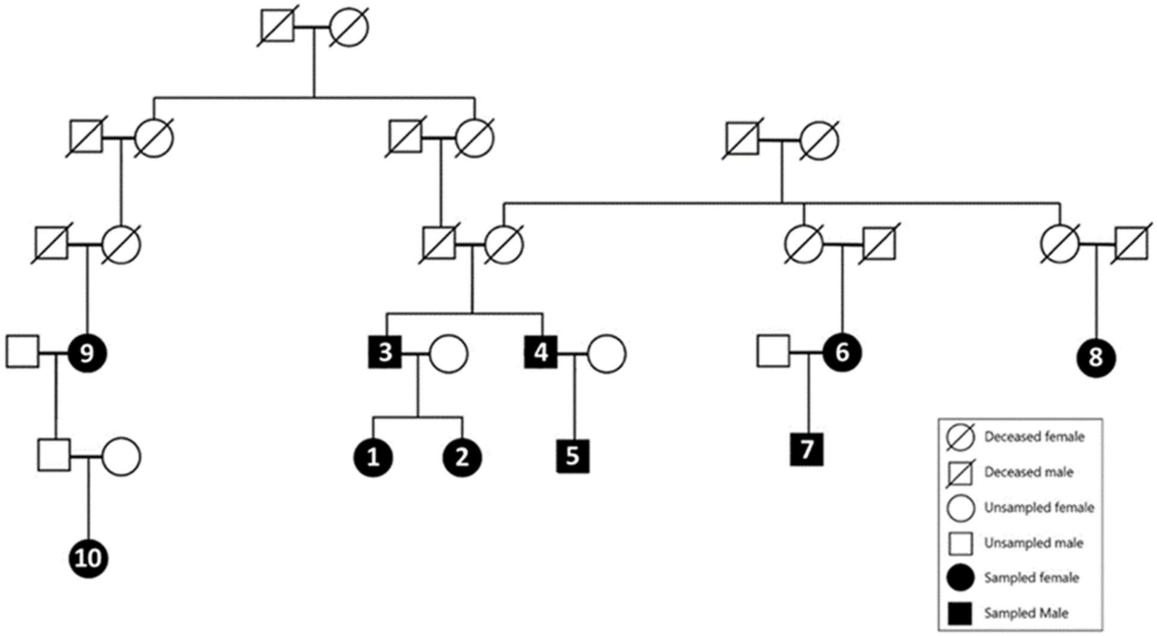

Table S1. The genetic relationships shared by the family sample individuals from Figure S1.

Degree of kinship	Relationship	Pairs	Totals	
**1**	Parent/child	1, 3	4	6
		2, 3		
		4, 5		
		6, 7		
	Siblings	1, 2	2	
		3, 4		
**2**	Grandparent/grandchild	9, 10	1	4
	Uncle/nephew	3, 5	1	
	Uncle/niece	1, 4	2	
		2, 4		
**3**	First cousins	1, 5	7	7
		2, 5		
		3, 6		
		3, 8		
		4, 6		
		4, 8		
		6, 8		
**4**	First cousins once removed	1, 6	9	9
		1, 8		
		2, 6		

		2, 8		
		3, 7		
		4, 7		
		5, 6		
		5, 8		
		7, 8		
**5**	Second cousins	1, 7	5	5
		2, 7		
		3, 9		
		4, 9		
		5, 7		
**6**	Second cousins once removed	1, 9	3	3
		2, 9		
		5, 9		
**7**	Second cousins twice removed	3, 10	2	2
		4, 10		
**8**	Third cousins once removed	1, 10	3	3
		2, 10		
		5, 10		
**0**	Unrelated	6, 9	6	6
		6, 10		
		7, 9		
		7, 10		
		8, 9		
		8, 10		
	**Total**		45	

Algorithm S1. Pseudocode describing the workflow of the algorithm for identifying stretches of concordant SNP genotypes shares by a pair of individuals.

Input : A list of dataframes L_p, where each dataframe contains a pair of SNP profiles belonging to individuals i and j.

Output: A list of dataframes L_s, where each dataframe contains the locations of segments where the genotypes of individuals i and j are concordant with IBD.

function (L_p);
for *dataframe* in L_p **do**
    for each SNP n in *dataframe* **do**
        $G_{i,n} \leftarrow$ genotype of individual i at SNP n
        $G_{j,n} \leftarrow$ genotype of individual j at SNP n
        if $G_{i,n}$ is concordant with $G_{j,n}$ AND $G_{i,n+1}$ is concordant with $G_{j,n+1}$ AND $G_{i,n-1}$ is not concordant with $G_{j,n-1}$ **then**
            Start new segment.
            Store segment start location.
        end
        else if $G_{i,n}$ is concordant with $G_{j,n}$ AND $G_{i,n-1}$ is concordant with $G_{j,n-1}$ AND $G_{i,n+1}$ is not concordant with $G_{j,n+1}$ **then**
            End segment.
            Store segment end location.
        end
        Add segments shared by individuals i and j to L_s
    end
    return L_s
end

Algorithm S2. Pseudocode describing the workflow of the algorithm for window-based IBD segment detection.

```
Input  : A list of dataframes L_p, where each dataframe contains a pair
         of SNP profiles belonging to individuals i and j.
Output: A list of dataframes L_s, where each dataframe contains the
         locations of windows where the genotypes of individuals i and
         j are concordant with IBD.

function (L_p);
for dataframe in L_p do
    W_{i,j}
      ← Dataframe for storing windows belonging to individual i and j
    for each chromosome C in dataframe do
        W_c ← Dataframe for storing all windows in chromosome C
        m ← Index of the first SNP in chromosome C
        n ← Index of the last SNP in chromosome C
        for SNP index i in [m, ..., n − 60] do
            Calculate kinship coefficient for window from SNP_i to
            SNP_{i+60}
            if kinship coefficient > 0.23 then
                Store window start and end locations in dataframe W_c
            end
        end
        while window w in dataframe W_c overlaps with window w + 1
         do
            Merge windows w and w + 1
        end
        Store merged windows in dataframe W_{i,j}
    end
    for window w in dataframe W_{i,j} do
        Calculate proportion p of window w wherein at least one allele is
        shared per genotype
        if p < 0.95 then
            Remove window w from dataframe W_{i,j}
        end
    end
    Add remaining windows from W_{i,j} to L_s
end
return L_s
```

Table S2. Autosomal masked regions used in the identical by descent (IBD) detection and filtering algorithm, which also represent gaps in single nucleotide polymorphism (SNP) coverage in the Verogen® ForenSeq Kintelligence Kit.

Chromosome	Start SNP	End SNP	Start position (Mbp)	End position (Mbp)
**1**	rs481357	rs6670984	120200843	145561594
**2**	rs1016839	rs1724120	88770768	96809331

**3**	rs7649188	rs7630912	88978538	94452002
**4**	rs4599384	rs1459814	48318390	53239603
**5**	rs1011814	rs6898102	44335820	49874330
**6**	rs7760349	rs6934646	57112128	64693014
**7**	rs10245203	rs4422672	57217249	62203031
**8**	rs6988483	rs1828494	42351311	50054112
**9**	rs7037234	rs449851	38771831	70984372
**10**	rs2505160	rs2744069	37309400	43374226
**11**	rs11038993	rs1792509	46810916	56670810
**12**	rs10772019	rs10506125	33085304	39120287
**16**	rs4889673	rs520151	31580272	48079134
**18**	rs786002	rs8099549	14914854	19826742
**20**	rs2076559	rs7271152	25187213	30506673

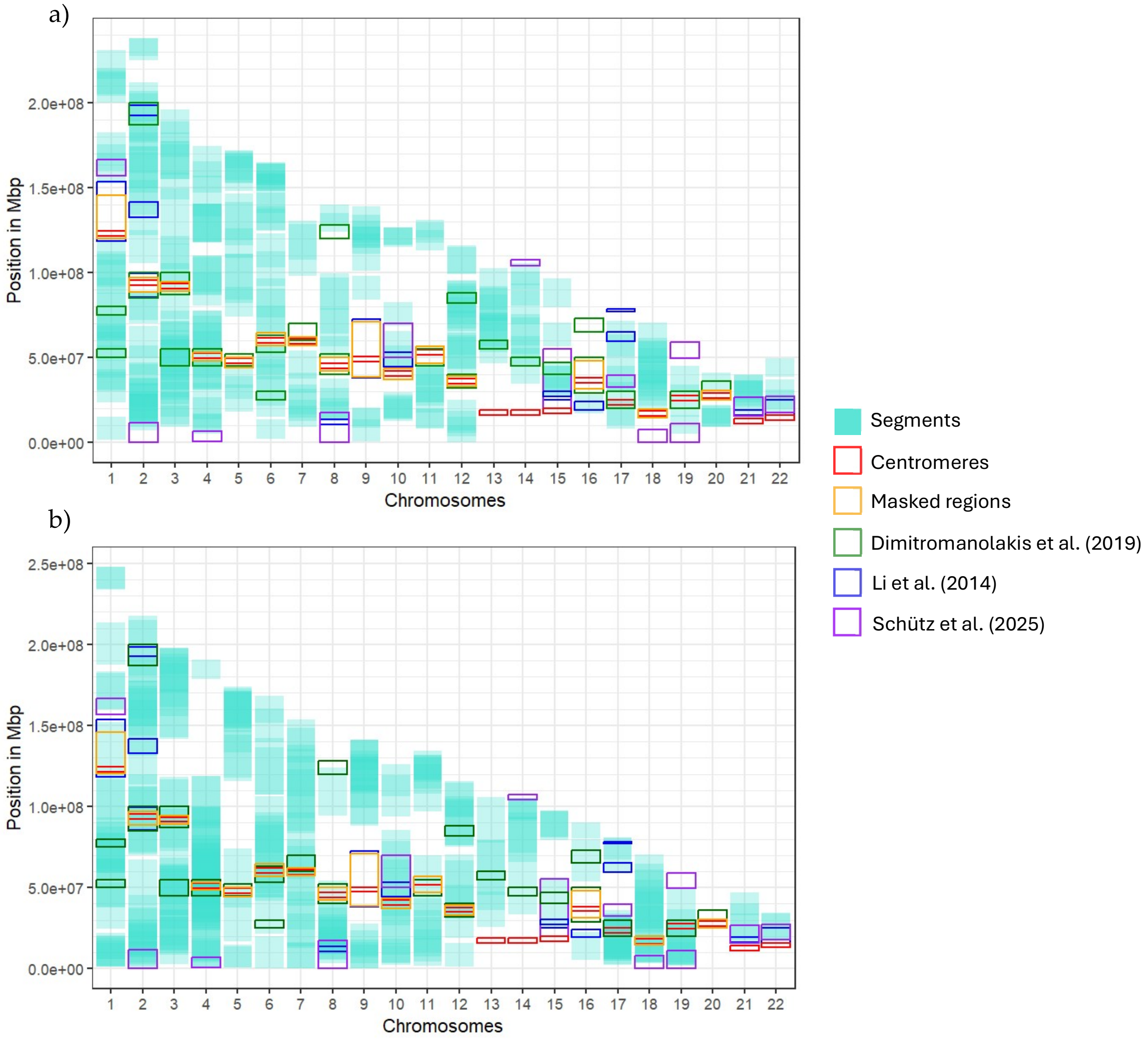

Figure S2. All segments shared by unrelated test individuals for Algorithm S1 (a) and Algorithm S2 (b), mapped alongside the centromeres, increased sharing regions identified in the literature and masked regions used in the IBD segment detection and filtering algorithm [34,46,48].

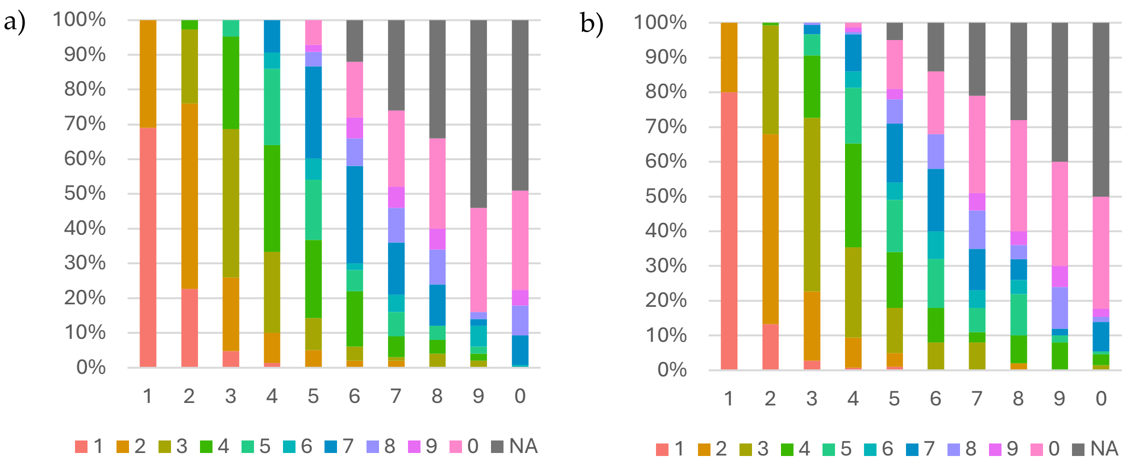

Figure S3. Results for the gamma distribution method of kinship inference for Algorithm S1 (a) and Algorithm S2 (b). 'NA' results indicate pairs whose relationship could not be inferred due to sharing less than two segments.

Table S3. Additional masked regions applied to the unrelated test pairings, which were identified by mapping segments shared by these pairs.

Chromosome	Start SNP	End SNP	Start position (Mbp)	End position (Mbp)
**2**	rs12471545	rs4665408	12168075	28521057
**3**	rs6599132	rs12715576	41039907	62044326
**4**	rs7672883	rs6554464	38006686	58296826
**6**	rs12210237	rs4708866	151639599	158022745
**12**	rs771655	rs1405780	77545429	90128655
**13**	rs9540208	rs9573824	65335655	76833684
**18**	rs11081813	rs1787531	31075341	47293738
**20**	rs3761161	rs2076584	10110237	19970705
**21**	rs6516727	rs2222956	27525506	37101040

References

1. Watson, J.L.; Grisedale, K.; Coakley, L.; McNevin, D.; Ward, J. Extended Kinship Inference Part 1: Evaluation of Short Tandem Repeats and Single Nucleotide Polymorphisms Using Likelihood Ratios and Haplotype Matching. *Forensic Genom.* **2025**, *5*, 13–31. https://doi.org/10.1089/forensic.2025.0001.
2. Schütz, O.; Maróti, Z.; Tihanyi, B.; Kiss, A.P.; Nyerki, E.; Gînguță, A.; Kiss, P.; Varga, G.I.B.; Kovács, B.; Maár, K.; et al. Unveiling the origins and genetic makeup of the "forgotten people": A study of the Sarmatian-period population in the Carpathian Basin. *Cell* **2025**, *188*, 4074–4090.e4011. https://doi.org/10.1016/j.cell.2025.05.009.
3. Li, H.; Glusman, G.; Hu, H.; Shankaracharya; Caballero, J.; Hubley, R.; Witherspoon, D.; Guthery, S.L.; Mauldin, D.E.; Jorde, L.B.; et al. Relationship estimation from whole-genome sequence data. *PLoS Genet.* **2014**, *10*, e1004144. https://doi.org/10.1371/journal.pgen.1004144.
4. Dimitromanolakis, A.; Paterson, A.D.; Sun, L. Fast and Accurate Shared Segment Detection and Relatedness Estimation in Un-phased Genetic Data via TRUFFLE. *Am. J. Hum. Genet.* **2019**, *105*, 78–88. https://doi.org/10.1016/j.ajhg.2019.05.007.
